# Supplementary material for: Predicting liver ablation volumes with real-time MRI thermometry
Source: JHEP Rep. 2024 Aug 31;6(11):101199. doi: 10.1016/j.jhepr.2024.101199 (PMC11686057; doi:10.1016/j.jhepr.2024.101199)
Supplement: Multimedia component 1 [file mmc1.pdf]

Prediction of ablation volume using real-time thermometry in MRI-guided microwave ablation of liver lesions

Osman Öcal, Olaf Dietrich, Sergio Lentini, Pierre Bour, Thibaut Faller, Valery Ozenne, Florian Maier, Matthias Philipp Fabritius, Daniel Puhr-Westerheide, Vanessa F. Schmidt, Elif Öcal, Ricarda Seidensticker, Moritz Wildgruber, Jens Ricke, Max Seidensticker

Table of contents

Fig. S1……………………………………………………………...…………………….2

Fig. S2……………………………………………………………..…….………………..8

Supplementary video legend…………………………………………………………11

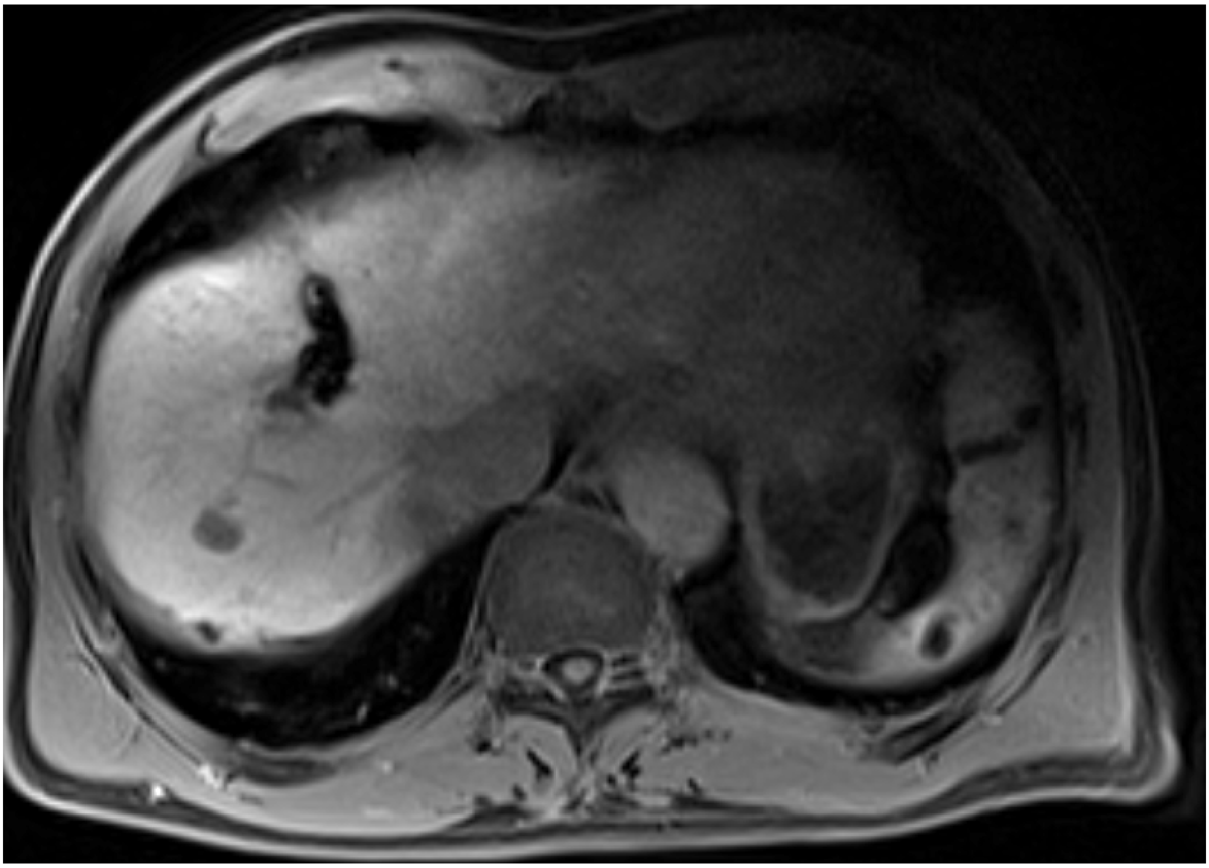

Fig. S1a. A 15x11 mm lesion is seen in Segment 7. There are artifacts related to the previous surgical materials in Segment 8.

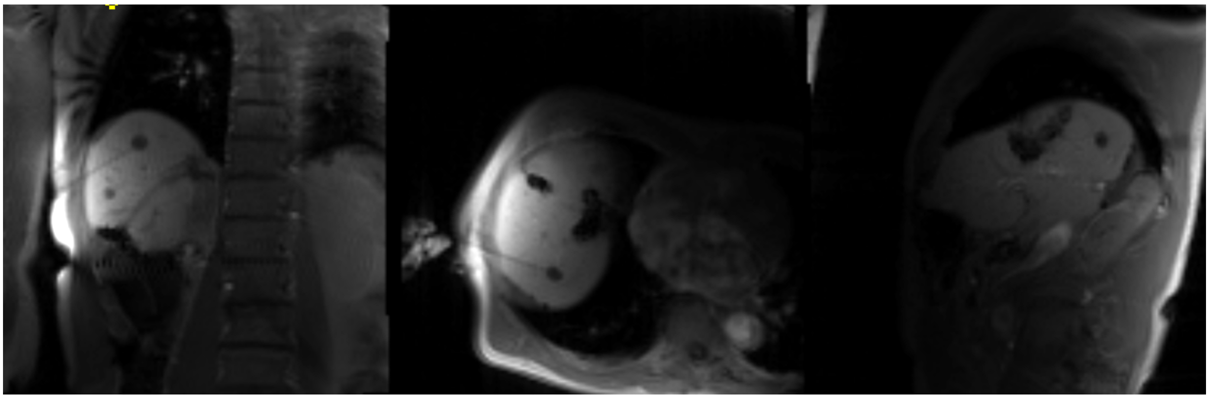

Fig. S1b. Needle placement under fluoroscopic T1w GRE sequence (BEAT interactive) in 3 perpendicular slice orientations.

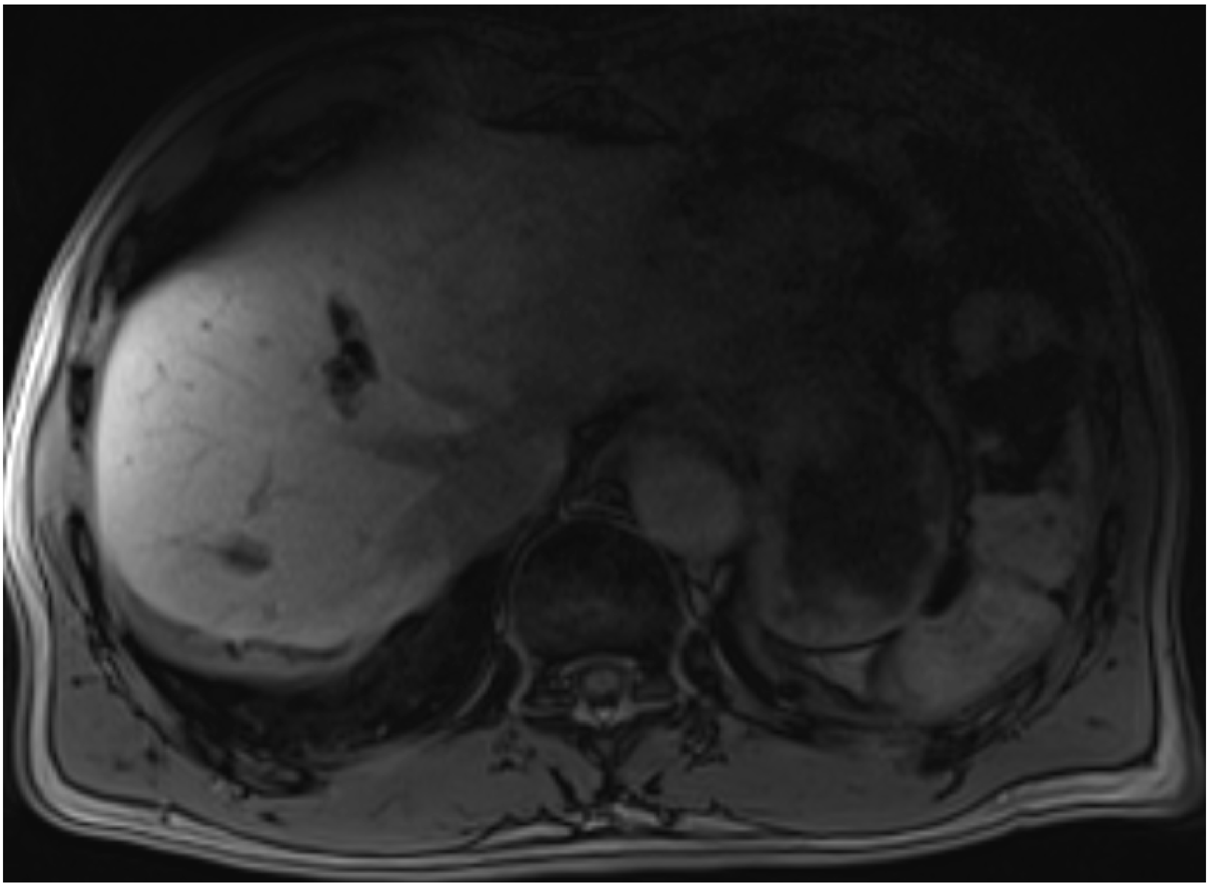

Fig. S1c. Needle position was confirmed before initation of energy deposition.

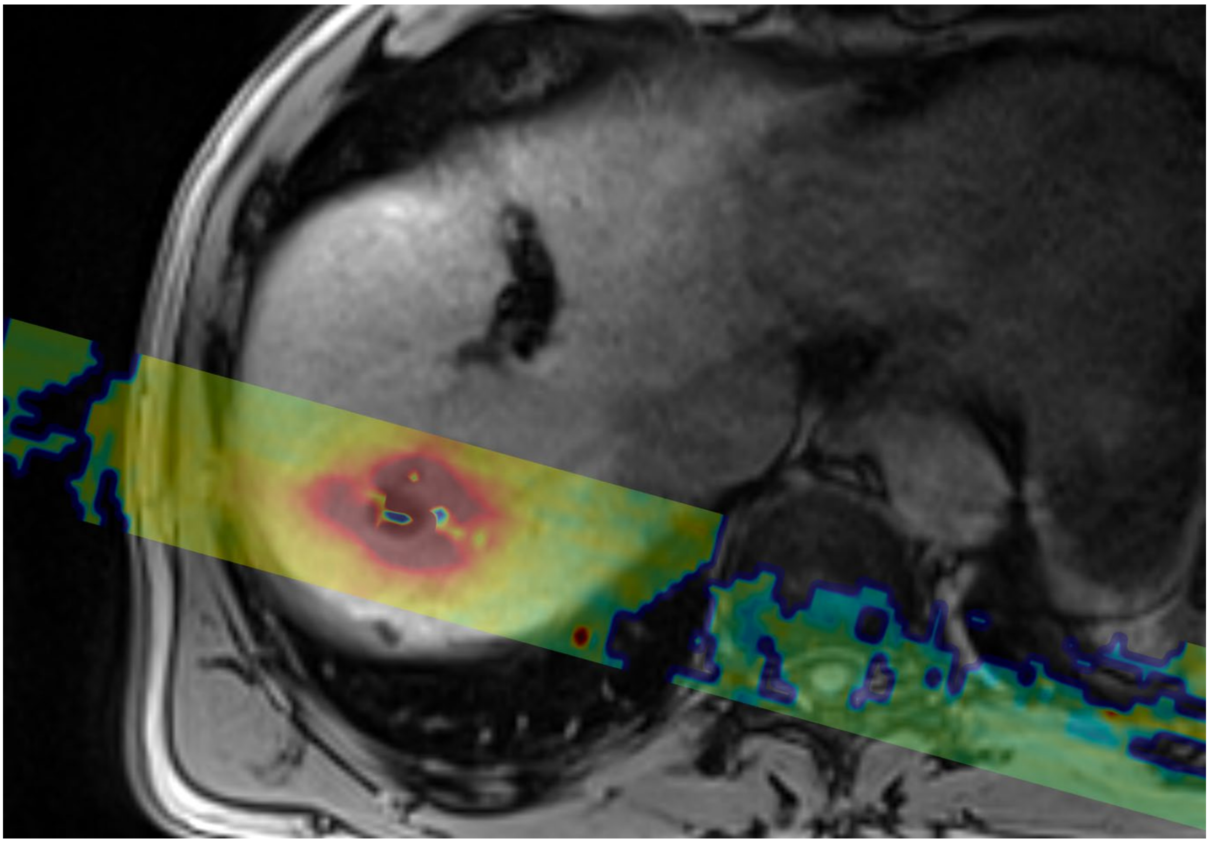

Fig. S1d. Temperature map shows satisfactory ablation volume with sufficient ablation margin in all directions.

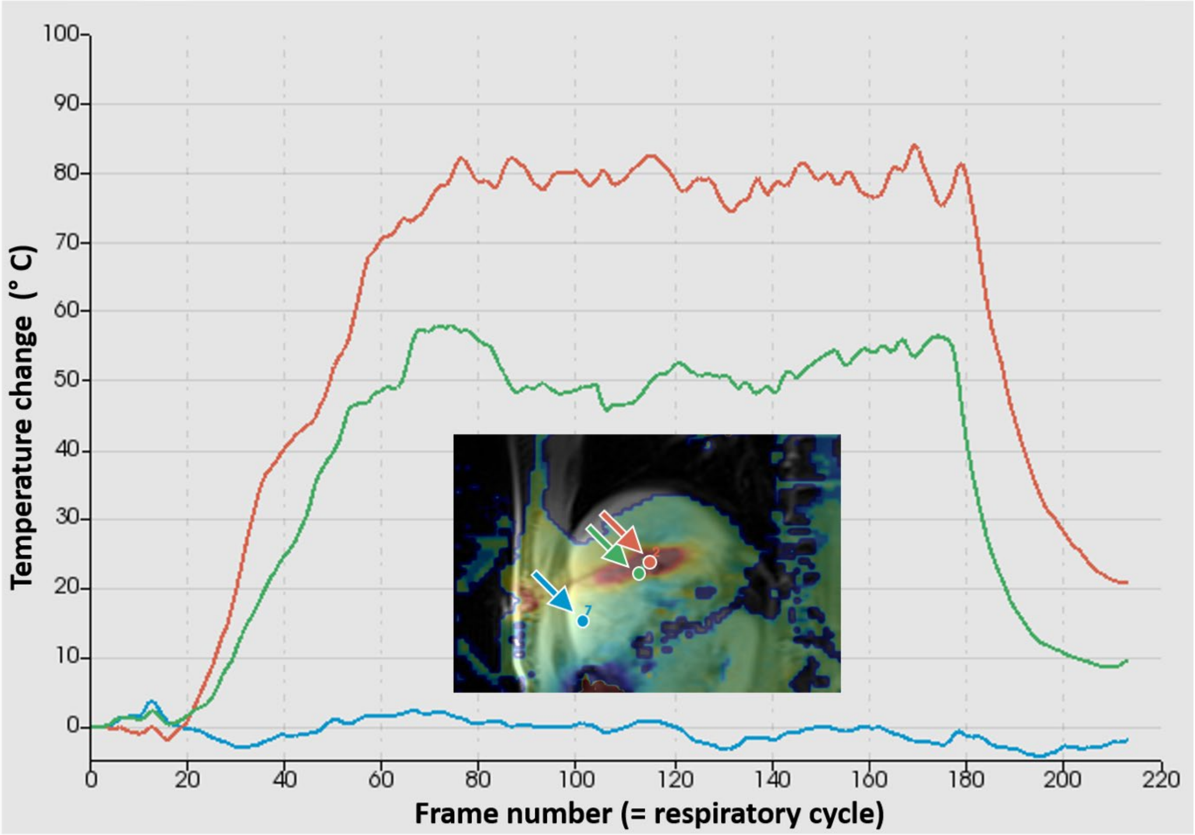

Fig. S1e. Temperature-time curve of an ablation shows temperature change at three different points within the liver. Red represents a point in the center of the lesion, green periphery, and blue a point in the liver away from the ablation area. Temperature drop within the ablation area can be seen after the completion of the energy deposition.

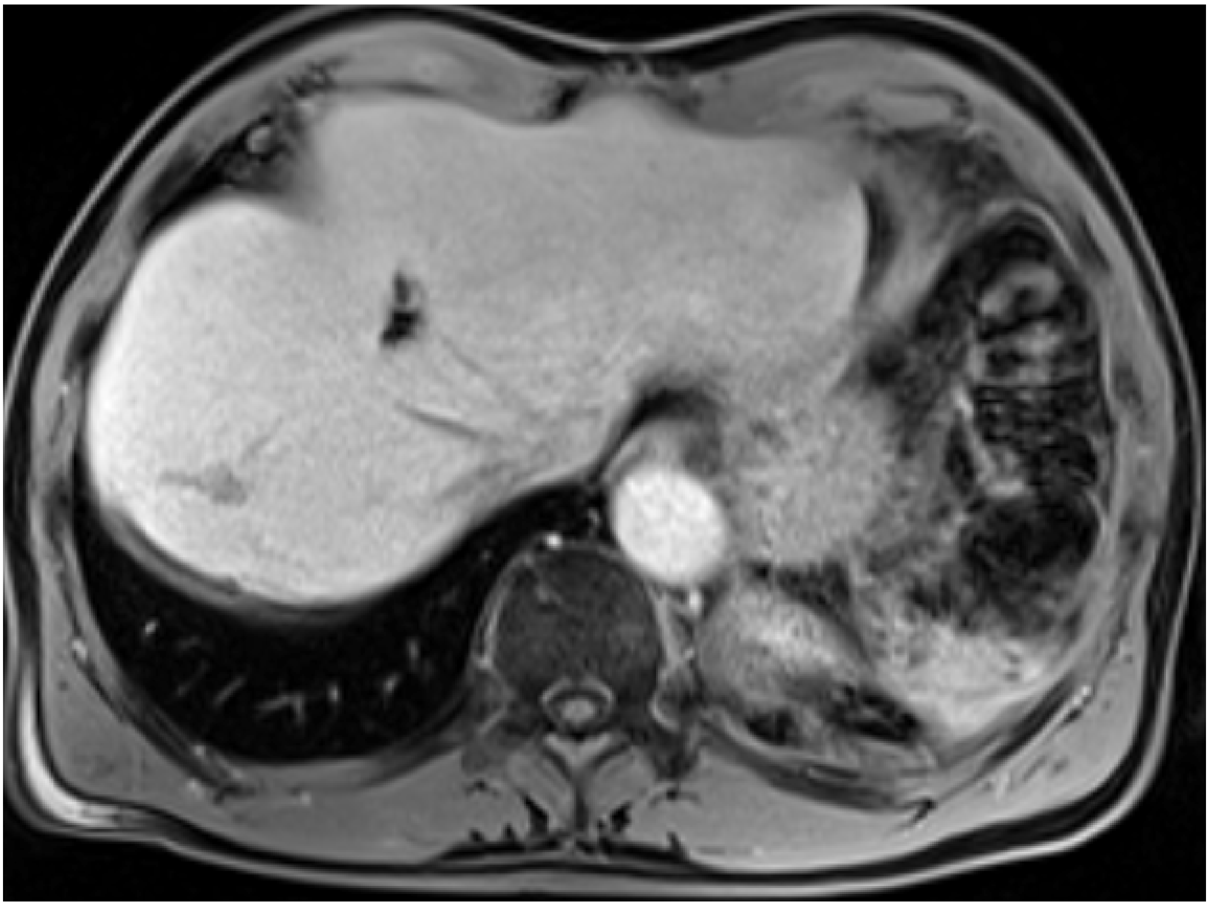

Fig. S1f. Follow-up MRI image 18 months after the treatment shows no sign of local residual disease.

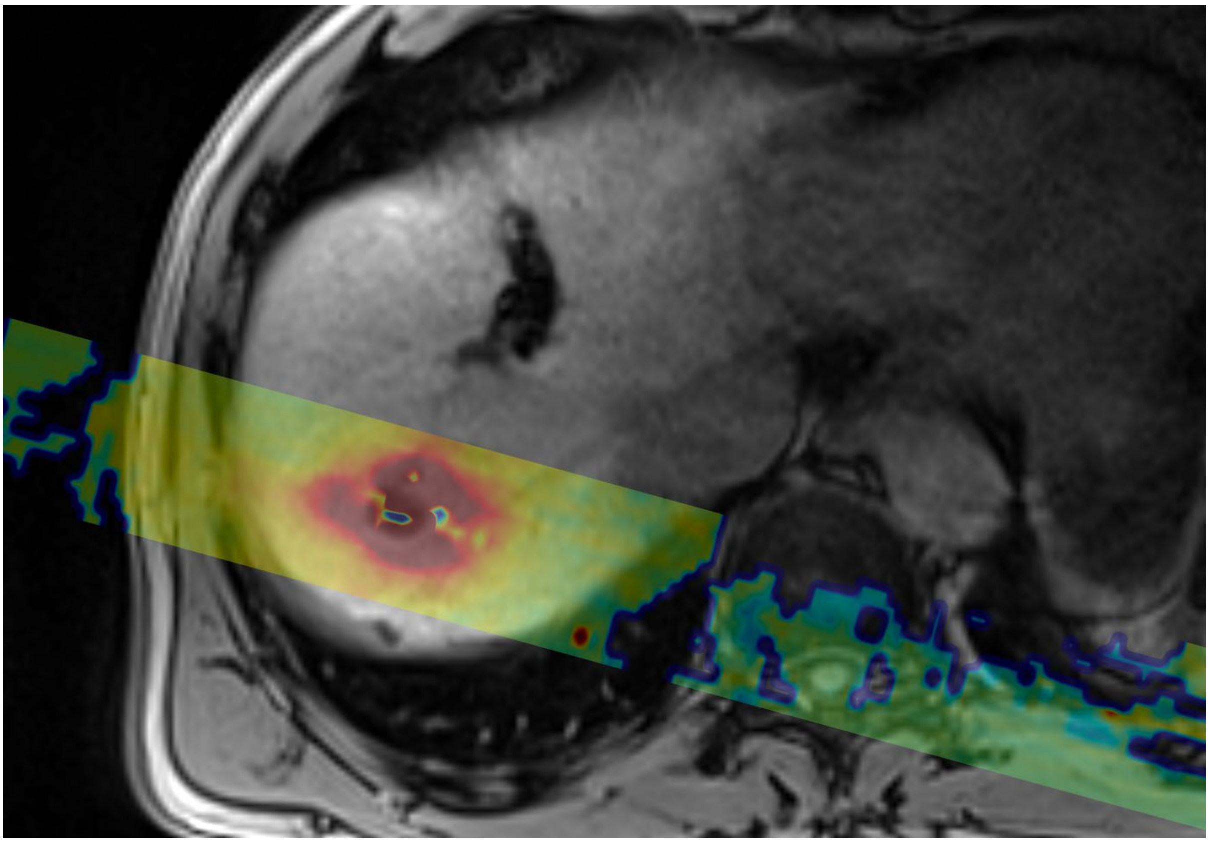

Fig. S2a. Temperature map shows no thermometry-related artifacts and no apparent movement related artifacts. The lesion can be differentiated as darker area within the red area. Both thermometry (signal-to-noise) and movement-related artifact score were graded as 1 (perfect).

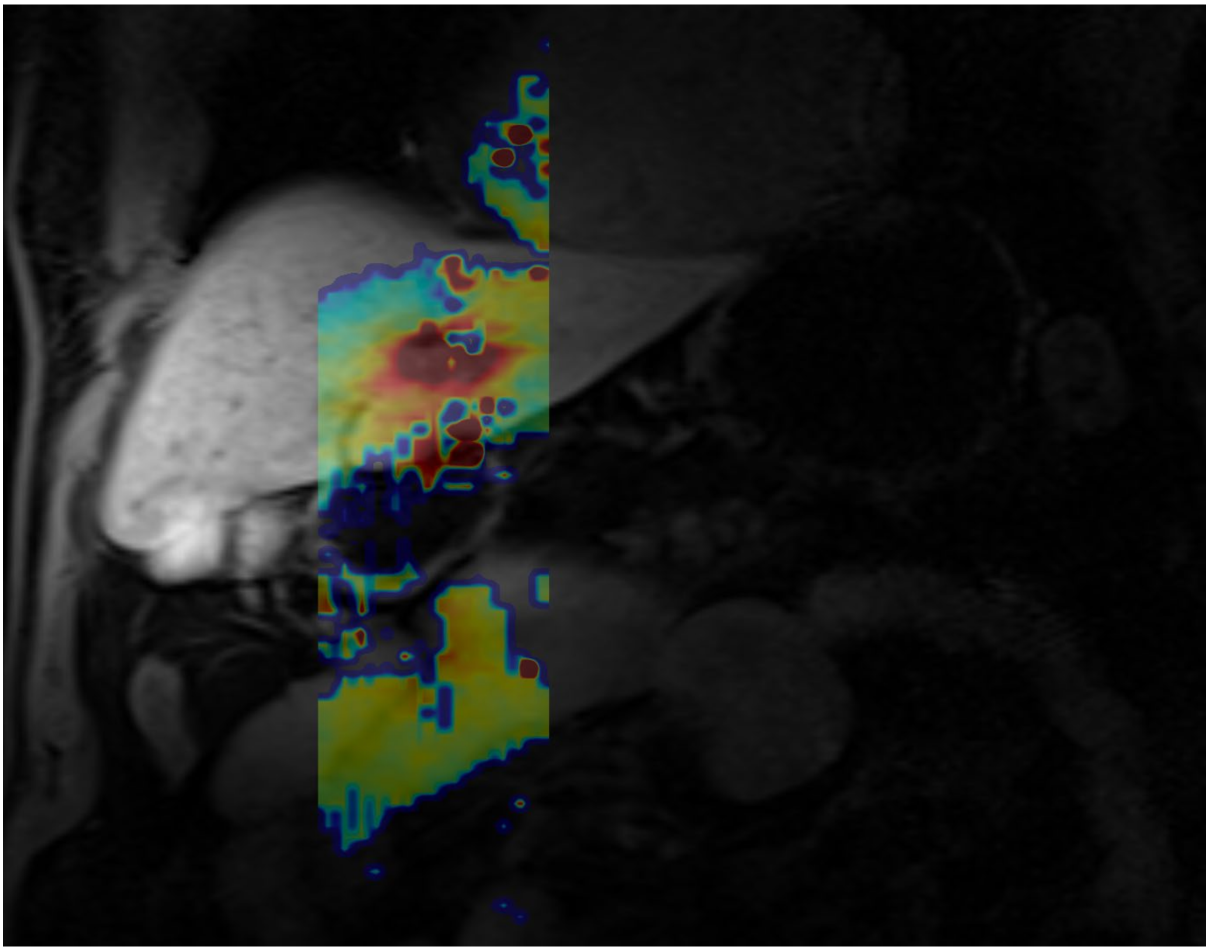

Fig. S2b. Temperature map shows good heating around the lesion, and the main thermal dose island (arrowhead) can be differentiated from movement-related artifacts reaching beyond the liver. Thermometry (signal-to-noise) score was graded as 3 and movement-related artifact as 2.

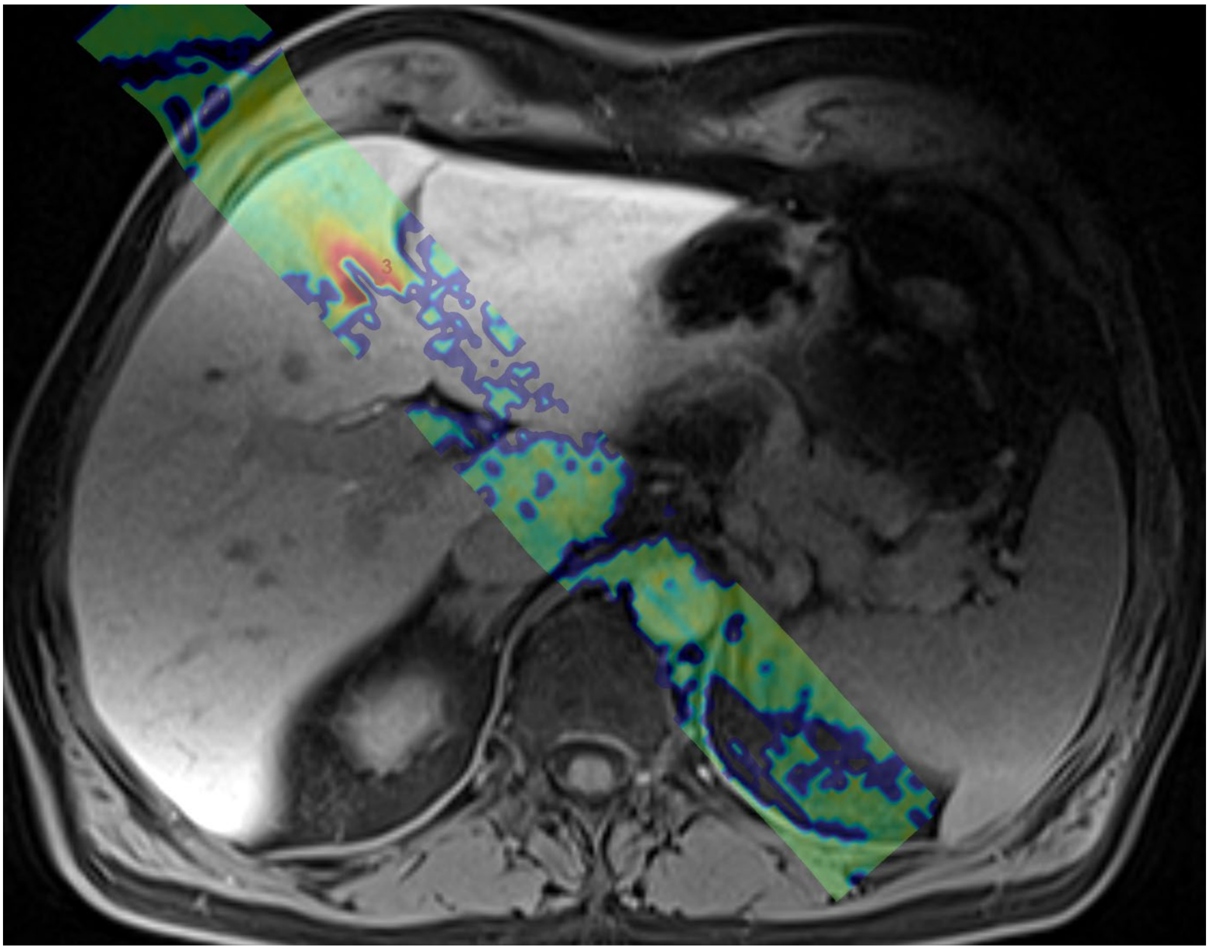

Fig. S2c. Temperature map shows heating at the superficial part of the lesion; however, no signal is received from the other parts of the lesion. The artifact related to the needle can be seen within the red area. Thermometry (signal-to-noise) score was graded as 5, and this lesion has been excluded from further analysis.

Supplementary video legend.

Real-time thermometry of an exemplary case. After the starting of energy deposition, thermometry maps visualize the temperature within the image acquisition area on a scale of green to red according to the temperature. After cessation of the energy deposition (starting from the 14th second), the temperature diffusion can be seen as shrinkage of the red areas.
