# Supplementary material for: Predicting liver ablation volumes with real-time MRI thermometry
Source: JHEP Rep. 2024 Aug 31;6(11):101199. doi: 10.1016/j.jhepr.2024.101199 (PMC11686057; doi:10.1016/j.jhepr.2024.101199)
Supplement: Multimedia component 5 [file mmc5.pdf]

Research article

JHEP Reports

Predicting liver ablation volumes with real-time MRI thermometry

Authors

Osman Öcal, Olaf Dietrich, Sergio Lentini, …, Moritz Wildgruber, Jens Ricke, Max Seidensticker

Correspondence

osman.oecal@med.uni-muenchen.de (O. Öcal).

Graphical abstract

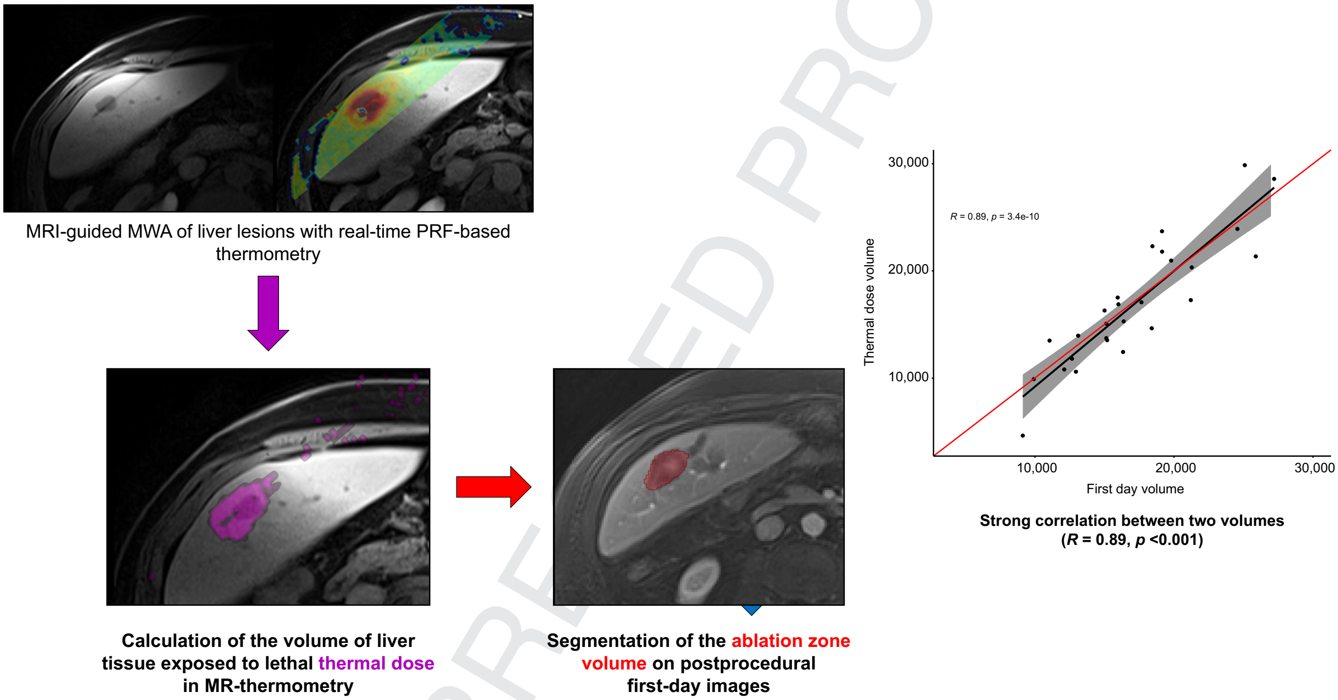

Highlights:

- Evaluation of 30 patients who underwent microwave ablation with the acquisition of MR thermometry.
- In 81.9% of the lesions, real-time thermal dose estimation was possible without artifacts limiting image interpretation.
- Volumes of thermal dose estimations strongly correlated with the ablation zone volume images on first procedural day (R = 0.89, p <0.001).

Impact and implications:

Heat-based ablation is an established treatment for liver tumors; however, there is a considerable rate of incomplete treatment because of the lack of real-time visualization of the treated area during treatment. Our results show that MRI-guided ablation enables the visualization of the treatment area in real-time with high accuracy using a special technique of MR thermometry in patients with liver tumors.

UNCORRECTED PROOF

https://doi.org/10.1016/j.jhepr.2024.101199

© 2024 The Author(s). Published by Elsevier B.V. on behalf of European Association for the Study of the Liver (EASL). This is an open access article under the CC BY license (http://creativecommons.org/licenses/by/4.0/).

Predicting liver ablation volumes with real-time MRI thermometry

Osman Öcal[1,2,*], Olaf Dietrich[1], Sergio Lentini[1], Pierre Bour[3], Thibaut Faller[3], Valery Ozenne[3], Florian Maier[4], Matthias Philipp Fabritius[1], Daniel Puhr-Westerheide[1], Vanessa F. Schmidt[1], Elif Öcal[1], Ricarda Seidensticker[1], Moritz Wildgruber[1], Jens Ricke[1], Max Seidensticker[1]

JHEP Reports **2024**. vol. 6 | 1–6

Check for updates

Background & Aims: MRI guidance offers better lesion targeting for microwave ablation of liver lesions with higher soft-tissue contrast, as well as the possibility of real-time thermometry. This study aims to evaluate the correlation of real-time MR thermometry-predicted lesion volume with the ablation zone in postprocedural first-day images.

Methods: This single-center retrospective analysis evaluated prospectively included patients who underwent MRI-guided microwave ablation with real-time thermometry between December 2020 and July 2023. All procedures were performed under general anesthesia on a 1.5 T MRI scanner. Real-time thermometry data were acquired using multi-slice gradient-echo echoplanar imaging sequences, and thermal dose maps (CEM43 of 240 min as a threshold) were created. The volume of tissue exposed to a lethal thermal dose in MR thermometry (thermal dose) was compared with the ablation zone volume in portal phase T1w MRI on the postprocedural first day using the Pearson correlation test, and visual quantitative assessment by radiologists was performed to evaluate the similarity of shapes and volumes.

Results: Out of 30 patients with 33 lesions with thermometry images, six (18.1%) lesions were excluded because of artifacts limiting interpretation of thermal dose volume. Twenty-four patients with 27 lesions (20 male, age 63.1 ± 9.1 years) were evaluated for the volume correlation. The volume of thermal dose-predicted lesions and the postprocedural first-day ablation zones showed a strong correlation ($R = 0.89$, p <0.001). Similarly, visual similarity of molecular resonance thermometry-predicted shape and the ablation zone shape was graded as perfect in 23 (85.1%) lesions.

Conclusions: Real-time thermal dose-predicted volumes show very good correlation with the ablation zone volumes in images obtained 1 day after the procedure, which could reduce the local recurrence rates with the possibility of re-ablating lesions within the same procedure.

© 2024 The Author(s). Published by Elsevier B.V. on behalf of European Association for the Study of the Liver (EASL). This is an open access article under the CC BY license (http://creativecommons.org/licenses/by/4.0/).

Introduction

Local ablation, particularly thermal ablation, is the main pillar of therapy in patients with oligometastatic disease or early-stage hepatocellular carcinoma. Guidelines on many disease modalities recommend thermoablation, such as radiofrequency ablation or microwave ablation (MWA), as first-line treatment according to the number and size of the lesions.[1,2]

Interventional thermoablation procedures aim at destroying pathological tissues via a localized energy deposit. The ablation zone is aimed to cover the lesion borders at least 0.5 cm in each direction while avoiding damage to surrounding at-risk structures.

However, during the energy deposition of thermoablation, whatever the modality and the medical device considered, it is rarely possible to objectify the energy sent during the ablation. In most procedures, ablation zone estimation is done based on the empirical parameters suggested by the manufacturer (power and emission time), which is usually determined in *ex vivo* experiments without the influence of perfusion and not on the actual effect obtained locally in the targeted tissue. Hence, customization of the treatment is impossible, and the prediction of the ablation zone is not precise. As a result, thermal ablation procedures are associated with a 6–12% local recurrence as a result of incomplete lesion coverage despite the acquisition of contrast-enhanced control images at the end of the procedures,[3] in which the unenhanced volumes represents the ablation zone.[4] Further on, application in proximity to heat-sensitive structures is restricted. Maneuvers such as saline injection could be used to protect neighboring structures, but they are not possible in some locations, or their efficacy cannot be confirmed during the procedure.

Thus, real-time monitoring of thermal energy deposition is necessary to improve lesion coverage of the ablation zone as well as to avoid heat-induced complications. Molecular resonance imaging (MRI) guidance improves lesion targeting with high soft-tissue contrast and also allows non-invasive

* Corresponding author. Address: Department of Radiology, University Hospital, Ludwig Maximilian University of Munich, Marchioninistrasse 15, 81377 Munich, Germany. Tel.: +49-4400-72750.
E-mail address: osman.oecal@med.uni-muenchen.de (O. Öcal).
https://doi.org/10.1016/j.jhepr.2024.101199

monitoring and visualizing the temperature in real time. However, up to now, monitoring of the thermoablation procedures in the liver has been limited because of breathing-related movements and ablation device-related artifacts.

This study aims to evaluate the correlation between the molecular resonance (MR) thermometry-predicted lesion volume obtained by real-time 3D temperature mapping during MWA ablation of liver tumors with the ablation zone detected as unenhanced volume in portal venous phase in post-procedural first-day contrast-enhanced MRI images.

Patients and methods

We performed a retrospective study of all patients who underwent MRI-guided MWA procedures with MRI thermometry sequence acquisition in our center between December 2020 and July 2023. The analysis was approved by the ethics committee of our hospital, and informed consent was waived because of the retrospective nature of the study. All patients gave informed consent for the procedure, and were recruited prospectively to a trial on sequence and workflow evaluation of the interventional MRI software package of the vendor after written informed consent.

A total of 35 patients with 40 lesions underwent MRI-guided MWA with thermometry sequence acquisition during the study period. All treatment decisions were made based on a multidisciplinary tumor board. Four lesions were excluded owing to two rounds of ablation for the same lesion (overlapping coagulation zones), one lesion because of needle movement during ablation by the operator, and two lesions because of patient movement related to anesthesia depth which resulted in artifacts on thermometry map frames. In total 30 patients with 33 lesions were included for further analysis (see the flow chart in Fig. 1).

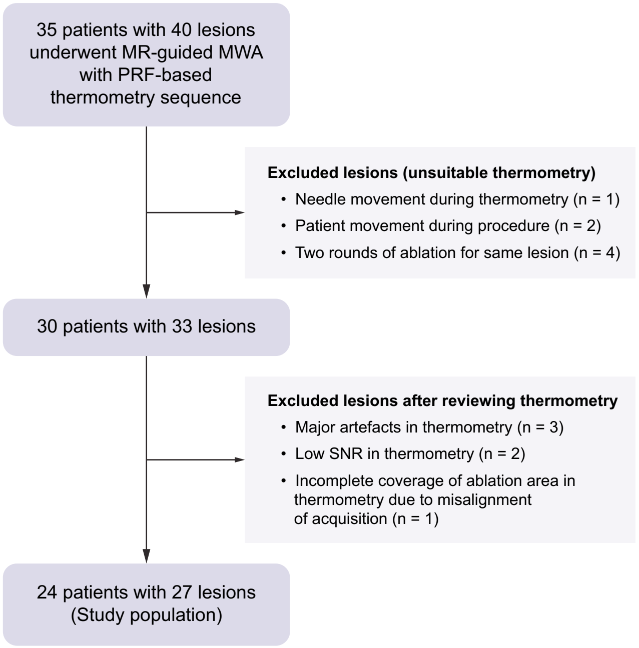

Fig. 1. Flow chart. n refers to lesion number.

Ablation procedure

All procedures were done by the same senior radiologist with 18 years of experience in image-guided interventions under general anesthesia in a closed whole-body 1.5 tesla (T) scanner (MAGNETOM Aera; Siemens Healthineers, Erlangen, Germany) with a short bore design and the XQ gradient system (max. amplitude 45 mT/m, max. slew rate 200 T/m/s). Anesthesia induction was done in the preparation room next to the MRI, so the scanner was not occupied during this period. A receive-type surface loop coil (Siemens Healthineers, Erlangen, Germany) with a diameter of 110 mm and 12 coil elements of the built-in spine array were used. MWA was performed using MR conditional microwave systems (AveCure, MedWaves, San Diego, CA or ECO-100A, ECO Medical Instruments Co., Ltd. Nanjing, China) and 14G microwave antenna with 1–4 mm active tip.

About 15 min after injecting weight-based (0.1 mL/kg body weight) hepatospecific contrast agent (Primovist, Bayer Vital, Leverkusen, Germany), axial and coronal T1-weighted (T1w) Dixon gradient echo (GRE) sequences were acquired (TR 6.8 ms, TE 2.4 and 4.8 ms, flip angle 10°, slice thickness 3 mm, field of view 380 × 380 mm^2, matrix 320 × 195, bandwidth 470 Hz/pixel, breath-hold, reconstruction of 'water' images) to localize the target lesion; the access route was planned using the interventional WIP package ASP 1428B 'LaserToTarget' (Siemens Healthineers). Sterile draping and local anesthesia were administered after defining the skin entry point with finger pointing. The microwave antenna was then placed under the guidance of a fluoroscopic T1w GRE sequence (BEAT interactive sequence with WIP package ASP 1075H 'Needle AutoAlign' [Siemens Healthineers]) for sequential imaging in three perpendicular slice orientations with TR 8.4 ms, TE 4.4 ms, flip angle 30°, matrix 128 × 128, frame rate 1/s). Following the placement of the antenna in the target lesion, the needle position was confirmed in two orthogonal diagnostic T1w Dixon GRE images (as described above). Then, proton-resonance-frequency (PRF)-based MR thermometry was performed with phase images acquired by a gradient-echo single-shot echoplanar imaging (EPI) sequence (C2P-research sequence). The EPI thermometry pulse sequence acquired 13 slices (3 mm slice thickness) with an in-plane resolution of 2.8 × 2.8 mm^2 and a field of view of 360 × 360 mm^2. The imaging plane was chosen paracoronal or parasagittal such that the microwave antenna was located in the central slice of the acquired block. In six patients, volumes (20 slices) were acquired during free breathing with TR of ~100 ms and a volume refresh rate of 2,000 ms. In this case, the MR-thermometry was computed using a motion correction algorithm combined with a principal component analysis of the phase variations.[5] In 27 patients, volumes were acquired during the resting state of the respiratory cycle, using the built-in respiratory gating system (thoracic belt), also with a 2,000 ms of acquisition window. The mean effective echo time, TE, was 18 ms, the flip angle was 90°, Grappa 2, and echo train length was 42. Partial-Fourier (factor 6/8) and parallel imaging (factor 2) were used to accelerate the acquisition; the receiver bandwidth was 1,445 Hz/pixel. The ablation procedure was started 60 s after the initiation of the MRI thermometry sequence. Based on the size of the lesions and depending on the MWA system used, the power and emission time or target temperature and emission time of

the ablation system were chosen according to the MWA manufacturer's recommendations. Thermometry measurements were continued for about 120 s after the completion of the ablation procedure. Thus, the total duration of the thermometry sequence was 3 min plus the time for the energy deposition of the ablation. An example case is presented in the supplementary material, Fig. S1 and Video 1.

Supplementary video related to this article can be found at https://doi.org/10.1016/j.jhepr.2024.101199.

The MRI phase and magnitude data were transmitted in real-time to a workstation to calculate temperature maps using the software 'Certis Solution' version 1.2.0 (Certis Therapeutics, Pessac, France).

Track ablation was performed in each procedure. Axial T2w fat-saturated turbo spin echo and T1w GRE sequences were obtained at the end of each treatment.

Postprocedural care and imaging

All patients were hospitalized for observation. MR images were obtained on a 1.5 T MR system (Magnetom Avanto, Magnetom Aera Siemens Healthcare, Erlangen, Germany) to evaluate the ablation zone 1 day after the procedure. MRI protocol included T2w axial HASTE and T1w GRE sequences with fat suppression (volumetric interpolated breath-hold examination; VIBE) before and 20, 50, and 120 s after intravenous injection of 0.1 ml/kg Gadobutrol (Bayer Vital, Leverkusen, Germany; 0.1 mmol/kg body weight).

Evaluation of thermal dose and ablation zone

Thermometry images were reviewed by two radiologists with 9 and 18 years' experience in liver ablations, and movement artifacts were graded using a scale of 1–5 by consensus, 1 being no artifacts and 5 being artifacts precluding image interpretation (Fig. S2). Similarly, signal-to-noise ratios were graded qualitatively by consensus of the two radiologists using the same scale, 1 being thermal signal warranting perfect visualization of the energy deposition and 5 being no thermal signal.

Then, using thermal dose images provided by the Certis Solution software, the volume of liver tissue exposed to lethal thermal dose (i.e. the volume with thermal dose above the CEM43 threshold of 240 min, which will be referred to as 'MR thermometry-predicted lesion' hereafter) was calculated automatically according to the empirical model of Sapareto.[6] The segmentation process started with the automatically identifying pixels with a thermal dose value over 240CEM. Then, the main thermal dose island covering the lesion around the microwave needle was selected manually, and any area reaching beyond the liver was excluded by comparing it with T1w images. Following this, the signal-free voxels occupied by the needle, which is encapsulated by the thermal dose, were manually added to the thermal dose. The resulting volume of the MR thermometry-predicted lesion was recorded.

Using the first-day images, the non-enhancing area in the portal phase, or when contrast agent was not used, the hyperintense area in T1w images was manually segmented, and its volume (referred to as 'ablation zone' below) was recorded. The qualitative correlation between the shape of the thermal dose and ablation zone in first-day images was graded with a scale of 1–5 by two radiologists, 1 being a perfect match and 5 being no match.

Statistical analysis

Analyses were performed using R statistical software (R version 3.6.3, R Foundation for Statistical Computing, Vienna, Austria). Categorical variables were reported as counts and percentages, and continuous variables as means and standard deviations or medians and interquartile ranges. The measurement agreement of MR thermometry-predicted lesion volume and the ablation zone volume in first-day images was evaluated using Bland–Altman analysis, and the Pearson correlation coefficient was calculated to compare the two volumes.

Results

Thermometry image quality

Thermometry images of a total of 30 patients with 33 lesions were reviewed. After reviewing the images, six cases were excluded because of significant artifacts: three had artifacts precluding appropriate thermal dose volume definition (diffuse thermometry signal; in one case because of surgical clips adjacent to the ablation zone), two had too low signal-to-noise ratio (thermometry quality score 5 for both), and one lesion because of incomplete coverage in thermometry imaging (owing to misalignment of thermometry acquisition and the lesion, Fig. 1).

Artifacts that precluded appropriate thermal dose evaluation were more frequent in the cases where thermometry was acquired without respiratory triggering (three out of six, 50%) as compared with cases with respiratory triggering (three out of 27, 11%; p = 0.057). There was no correlation between liver lobe and major artifacts (16.6% right lobe *vs.* 22.2% left lobe, p >0.99).

Final study cohort

In total, 24 patients (20 males, four females; age 63.1 ± 9.1 years) with 27 lesions were included in the final analysis (Fig. 1, Table 1). Twelve patients had hepatocellular carcinoma, six colorectal cancer, two neuroendocrine tumor of the pancreas, one adrenal, one prostate, one stomach, and one breast cancer metastasis. Twelve patients had underlying cirrhosis. Twenty lesions were in the right liver lobe, and seven in the left lobe. The mean lesion size was 13.2 ± 4.4 mm. The median procedure duration was 64 min (range 44–185 min) with a mean procedure duration per lesion of 54.4 ± 12.3 min.

Table 1. Baseline characteristics.

Parameter	Value
No. of patients	24
No. of lesions	27
Sex (male), n (%)	20 (83.3)
Age (years)	63.1 ± 9.1
Etiology, n	
Hepatocellular carcinoma	12
Colorectal cancer metastasis	6
Neuroendocrine tumor metastasis	2
Adrenocortical carcinoma metastasis	1
Prostate cancer metastasis	1
Stomach cancer metastasis	1
Breast cancer metastasis	1
Liver lobe, n	
Right	20
Left	7

Thermometry (signal-to-noise ratio) quality was scored as 1 in 15 lesions, 2 in eight lesions, and 3 in four lesions (mean 1.59 ± 0.74). Movement-related artifacts in thermometry images were scored as 1 in 18 lesions, 2 in eight lesions, and 3 in one lesion (mean 1.37 ± 0.56).

Correlation of MR thermometry prediction and ablation zone

In 25 lesions, the volume of the ablation zone was assessed in portal venous images. In one patient non-contrast images and in one patient portal venous phase computed tomography (CT) images were used.

Visual similarity of MR thermometry-predicted shape and the ablation zone shape was graded as 1 (perfect) in 23 (85.1%) lesions, 2 in three (11.1%) lesions, and 3 in one (3.7%) lesion (mean 1.18 ± 0.48).

The median MR thermometry-predicted lesion volume was 16,296 mm^3 (IQR, 13,502–21,155 mm^3) and the median ablation zone volume on first-day images was 16,338 mm^3 (IQR, 14,058–19,472 mm^3). The volume of thermal dose and ablation zone showed a strong correlation (R = 0.893 [95% CI, 0.778–0.950], p <0.001, Figs 2 and 3). Images of an example case is given in Fig. 4.

There was no significant difference (p = 0.65) in correlation coefficients between thermal dose and ablation zone for patients with underlying cirrhosis (0.910 [95% CI, 0.705–0.975]) and for patients without cirrhosis (0.869 [0.644–0.956]).

Discussion

Our results show that thermal dose predictions on PRF-based real-time MR thermometry correlate strongly with the ablation zone in first-day images. The visual assessment also showed a high agreement between the shape of the thermal dose prediction and the shape of the ablation zone.

Six (of 33, 18.1%) lesions were excluded from the analysis. Two lesions were excluded because of the low signal-to-noise ratio. In three lesions, thermal dose volume could not be calculated because of artifacts. In one of these three lesions, surgical clips adjacent to the ablation area were the main cause of the artifacts. In the last case, MR thermometry images did not cover the whole lesion. This case was the result of misalignment of imaging. One of the main advantages of GRE single-shot EPI is the possibility to cover up to almost 4 cm area by covering 13 slices and up to 20 slices in a TR of 2 s. This situation is a major improvement compared with previous thermometry sequences, which lead to partial visualization of the ablation volume owing to size constraints.

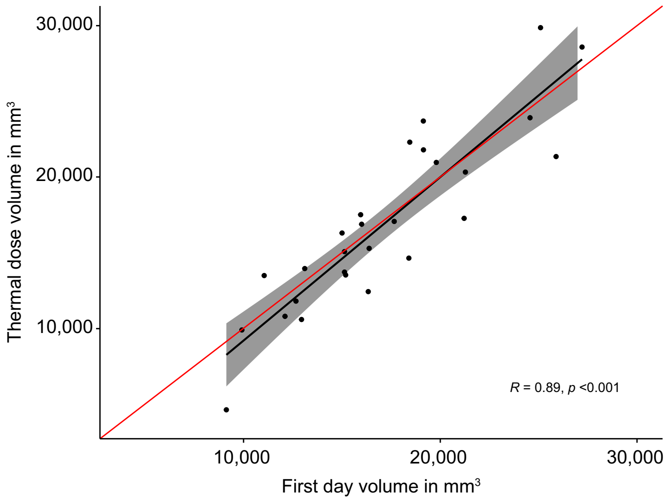

Fig. 2. Correlation plot of thermal dose volume and ablation zone volume at first day images in mm^3. The volume of thermal dose and ablation zone showed a strong correlation (R = 0.893 [95% CI, 0.778–0.950], p <0.001, Pearson correlation test). The red line shows the perfect correlation, and grey area represents the 95% confidence interval.

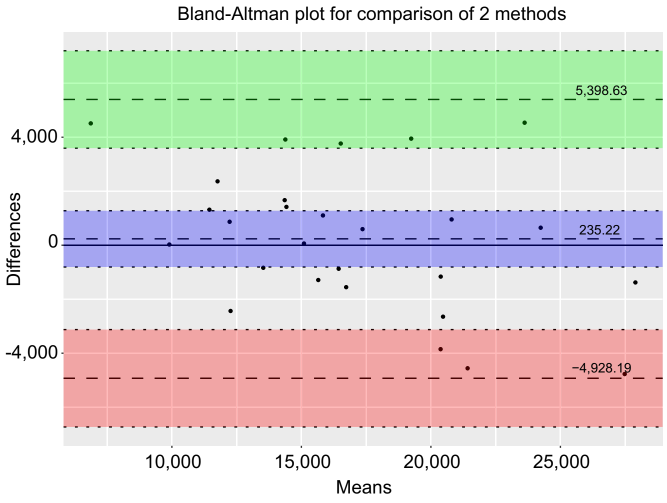

Fig. 3. Bland–Altman plot for MR thermometry-predicted lesion volume and ablation zone volume at first day images. The difference between MR thermometry-predicted lesion volume and ablation zone volume was plotted on the vertical axis and the mean of the two measurements was plotted on the horizontal axis. The solid (black) line represents zero line. The dashed line within magenta area represents the mean value for the data points and the dashed lines in green and red areas represents the 1.96 × standard deviations (95% CIs, Bland–Altman plot).

The main limitation of PRF-based MR thermometry is the motion sensitivity of the sequence, which limits its widespread usage in the liver.[7] It has been reported that the liver moves approximately 1–4 cm in the craniocaudal direction depending on the respiration depth.[8] Synchronization of the MR thermometry data acquisition to the respiratory cycle can eliminate movement-related phase artifacts and improve temperature maps.[9] Our results show that thermal maps of good quality can be obtained despite breathing movements either using retrospective motion-correcting post-processing of phase data acquired with sufficiently high temporal resolution (2 s per volume) or – preferably – using respiratory triggering.

To our knowledge, only one clinical study has tried to correlate real-time the MR thermometry-predicted lesion extent with the ablation zone, and there was no significant correlation between the two images.[10] However, radiofrequency ablation was used in that study, which is associated with more susceptibility and radiofrequency artifacts. In that study, 16 lesions were treated, but thermal dose could not be assessed because of artifacts in four (25%) lesions. This ratio was 15.6% in our study. Another study acquired temperature maps directly after to energy application (radiofrequency ablation) and demonstrated that temperature maps with 60 °C threshold level correlated better with the coagulation zone at 4 weeks, than

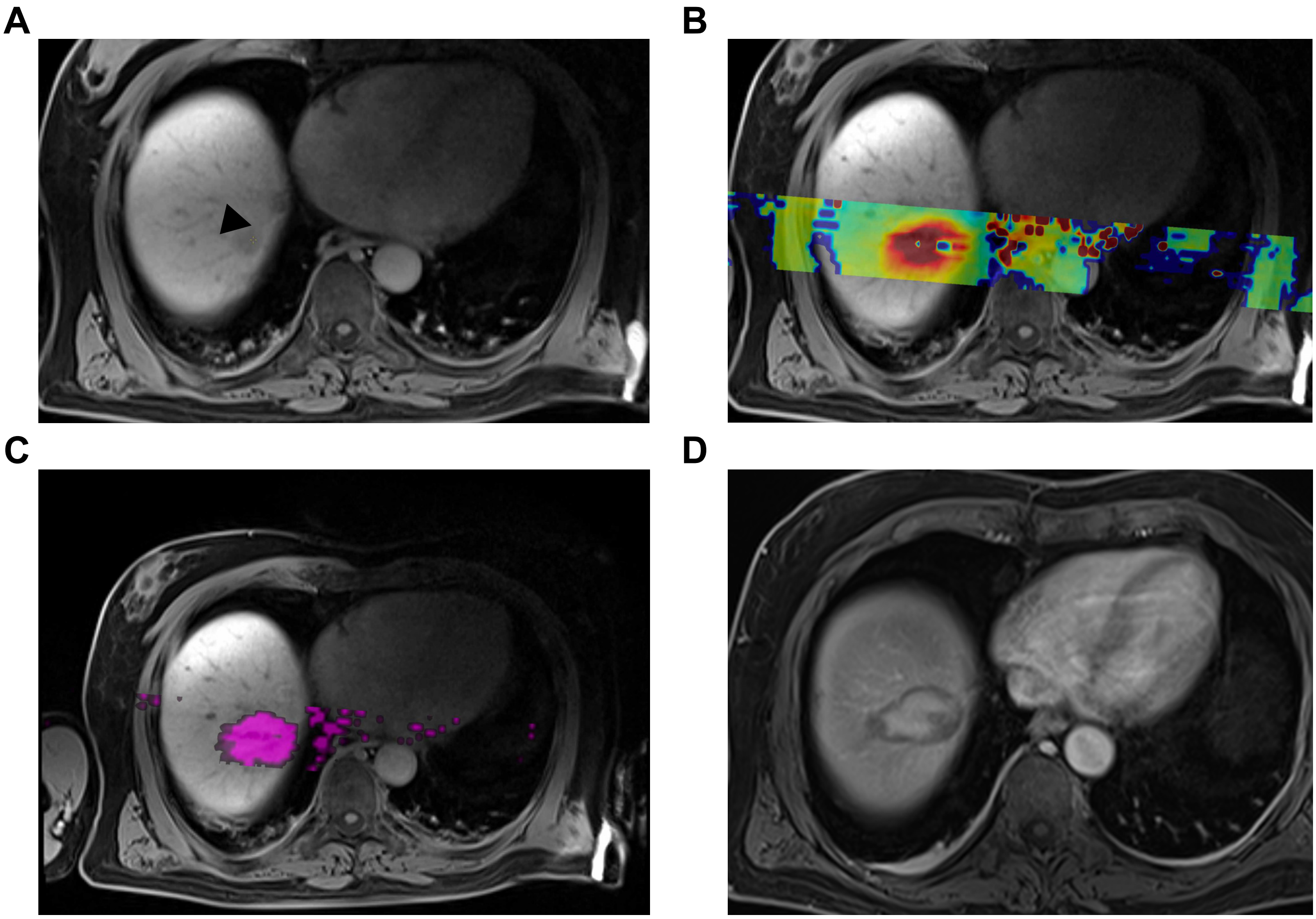

Fig. 4. Case example. (A) Axial T1w images after injection of gadoxetic acid shows a hepatocellular carcinoma lesion (arrowhead) in Segment 8. (B) Thermometry image shows MR-thermometry predicted temperature within the liver during the ablation. (C) Thermal dose image shows the volume exposed to lethal dose of thermal energy with a volume of 22,734 mm^3. (D) Portal phase MRI image on the postprocedural first day shows the ablation zone with a volume of 21,349 mm^3, which shows excellent similarity with thermal dose in terms of shape.

50 °C and 55 °C.[11] Although the images were obtained after energy deposition and, thus, a major cause of artifacts of the ablation device was not active during the thermometry sequences, 11/72 thermal maps were not of diagnostic quality. However, as temperature maps were obtained after thermoablation, this analysis considers only the temperature and not the duration of elevated temperatures. Also, since temperature starts to drop as energy deposition is stopped, measured temperatures do not represent the highest temperature reached during the ablation.

The microwave antenna was another source of artifact. Although no MRI signal could be obtained from the needle and in the directly adjacent voxels during the ablation, this did not interfere with monitoring the ablation edges. As the volume adjacent to this artifact zone was confined within the thermal dose area, it is natural to assume this area was also subjected to the thermal dose. To compensate for this situation, central parts with missing thermal information were included in the thermal dose volume as long as the outer border of the gross thermal dose volume enclosed these parts.

Local disease control is the ultimate aim of thermal ablation. Early detection of potential incomplete ablation is crucial, as in those patients, retreatment has to be performed as soon as possible to avoid local disease recurrence. After starting the ablation procedure, ultrasound guidance is not helpful in determining complete ablation because of the formation of gas bubbles and poor image contrast. Similarly, without contrast injection, evaluating the borders of the ablation zone and the lesion in CT-guided procedures is difficult. In addition to the possibility of real-time thermometry, MRI offers the advantage of better lesion visualization and, thus, targeting after the injection of a hepatospecific contrast agent.[12–14] Local recurrence is usually because of incomplete ablation at the edges of the ablation area, and using real-time thermometry, edges with suboptimal ablation margin (<5 mm) can be detected during the procedure, and re-ablation can be performed with no delay. In this study, ablation volume was detected using the contrast-enhanced images, which is used as gold standard because of better delineation of unvital liver parenchyma.[4] It has also been shown that MRI improves the detection of residual or recurrent lesions after thermal ablation compared with CT in primary or secondary liver tumors.[15,16] In addition, images obtained 1 day after the procedure has been used to evaluate the necrotic volume, because of early expansion of the ablation zone.[17]

This study has some limitations. Firstly, although patients were recruited prospectively, this was a retrospective subanalysis of the MR-guided interventions in our center. Second, some lesions had to be excluded from the study for

reasons such as needle position correction during ablation, or anesthesia-related patient movements, or two rounds of ablation, because it is currently not possible to combine thermal dose maps of multiple rounds of ablation in a single lesion (a situation that might be solved with updates in software). However, our study provides the first *in-vivo* correlation of real-time MR thermometry-predicted lesion volume and postprocedural (first day) ablation zone volumes.

In conclusion, the volume of tissue exposed to lethal thermal dose based on PRF-based real-time MR thermometry shows a very good correlation with the ablation zone volume in on postprocedural first day images. Real-time visualization of inadequate ablation margins could reduce the local recurrence rates with the possibility of re-ablating lesions within the same procedure. However, efforts to reduce the intricacy of MRI thermometry are needed to make this technique a robust routine approach in daily practice.

Affiliations

[1]*Department of Radiology, University Hospital, LMU Munich, Munich, Germany;* [2]*Department of Diagnostic and Interventional Radiology, University Hospital Heidelberg, Heidelberg, Germany;* [3]*Certis Therapeutics, Pessac, France;* [4]*Siemens Healthineers, Erlangen, Germany*

Abbreviations

CT, computed tomography; EPI, echoplanar imaging; GRE, gradient echo; MR, molecular resonance; MRI, molecular resonance imaging; MWA, microwave ablation; PRF, proton-resonance-frequency.

Financial support

No funding was received for this study.

Conflicts of interest

VO is co-founder and shareholder of Certis Therapeutics. TF and PB are Certis Therapeutics employees. FM is a Siemens Healthineers employee. JR and MS received lecture fees from Siemens Healthineers.

Please refer to the accompanying ICMJE disclosure forms for further details.

Authors' contributions

Conception and design of the study: OÖ, OD, JR, MS. Generation, collection, assembly, analysis and/or interpretation of data all authors. Drafting or revision of the manuscript: all authors. Approval of the final version of the manuscript: all authors.

Data availability statement

The data that support the findings of this study are not publicly available but are available from the corresponding author on reasonable request.

Supplementary data

Supplementary data to this article can be found online at https://doi.org/10.1016/j.jhepr.2024.101199.

References

Author names in bold designate shared co-first authorship

[1] Cervantes A, Adam R, Roselló S, et al. Metastatic colorectal cancer: ESMO Clinical Practice Guideline for diagnosis, treatment and follow-up. Ann Oncol 2023;34:10–32.

[2] Reig M, Forner A, Rimola J, et al. BCLC strategy for prognosis prediction and treatment recommendation: the 2022 update. J Hepatol 2022;76:681–693.

[3] Vietti Violi N, Duran R, Guiu B, et al. Efficacy of microwave ablation versus radiofrequency ablation for the treatment of hepatocellular carcinoma in patients with chronic liver disease: a randomised controlled phase 2 trial. Lancet Gastroenterol Hepatol 2018;3:317–325.

[4] Rempp H, Unterberg J, Hoffmann R, et al. Therapy monitoring of magnetic resonance-guided radiofrequency ablation using T1- and T2-weighted sequences at 1.5 T: reliability of estimated ablation zones. Invest Radiol 2013;48:429–436.

[5] Ozenne V, Toupin S, Bour P, et al. Improved cardiac magnetic resonance thermometry and dosimetry for monitoring lesion formation during catheter ablation. Magn Reson Med 2017;77:673–683.

[6] Sapareto SA, Dewey WC. Thermal dose determination in cancer therapy. Int J Radiat Oncol Biol Phys 1984;10:787–800.

[7] Kägebein U, Speck O, Wacker F, et al. Motion correction in proton resonance frequency-based thermometry in the liver. Top Magn Reson Imaging 2018;27:53–61.

[8] Rohlfing T, Maurer Jr CR, O'Dell WG, et al. Modeling liver motion and deformation during the respiratory cycle using intensity-based nonrigid registration of gated MR images. Med Phys 2004;31:427–432.

[9] Lu A, Woodrum DA, Felmlee JP, et al. Improved MR-thermometry during hepatic microwave ablation by correcting for intermittent electromagnetic interference artifacts. Phys Med 2020;71:100–107.

[10] Terraz S, Cernicanu A, Lepetit-Coiffé M, et al. Radiofrequency ablation of small liver malignancies under magnetic resonance guidance: progress in targeting and preliminary observations with temperature monitoring. Eur Radiol 2010;20:886–897.

[11] Rempp H, Hoffmann R, Roland J, et al. Threshold-based prediction of the coagulation zone in sequential temperature mapping in MR-guided radiofrequency ablation of liver tumours. Eur Radiol 2012;22:1091–1100.

[12] Rosenberg C, Jahn A, Pickartz T, et al. Gd-EOB-DTPA-enhanced MR guidance in thermal ablation of liver malignancies. PLoS One 2014;9:e109217.

[13] Fischbach F, Lohfink K, Gaffke G, et al. Magnetic resonance-guided freehand radiofrequency ablation of malignant liver lesions: a new simplified and time-efficient approach using an interactive open magnetic resonance scan platform and hepatocyte-specific contrast agent. Invest Radiol 2013;48:422–428.

[14] Fischbach F, Thormann M, Seidensticker M, et al. Assessment of fast dynamic imaging and the use of Gd-EOB-DTPA for MR-guided liver interventions. J Magn Reson Imaging 2011;34:874–879.

[15] Weiss J, Rempp H, Clasen S, et al. Diagnostic accuracy of different magnetic resonance imaging sequences for detecting local tumor progression after radiofrequency ablation of hepatic malignancies. Eur J Radiol 2017;94:85–92.

[16] Imai Y, Katayama K, Hori M, et al. Prospective comparison of Gd-EOB-DTPA-enhanced MRI with dynamic CT for detecting recurrence of HCC after radiofrequency ablation. Liver Cancer 2017;6:349–359.

[17] Alzubaidi S, Wallace A, Naidu S, et al. Single-arm prospective study comparing ablation zone volume between time zero and 24 h after microwave ablation of liver tumors. Abdom Radiol (NY) 2024;49:3136–3142.

Keywords:Microwave ablation; MRI; Thermometry; Thermal dose.

Received 3 April 2024; received in revised form 14 August 2024; accepted 21 August 2024; Available online 31 August 2024

Journal of Hepatology, Volume *6*

Supplemental information

Predicting liver ablation volumes with real-time MRI thermometry

Osman Öcal, Olaf Dietrich, Sergio Lentini, Pierre Bour, Thibaut Faller, Valery Ozenne, Florian Maier, Matthias Philipp Fabritius, Daniel Puhr-Westerheide, Vanessa F. Schmidt, Elif Öcal, Ricarda Seidensticker, Moritz Wildgruber, Jens Ricke, and Max Seidensticker

Prediction of ablation volume using real-time thermometry in MRI-guided microwave ablation of liver lesions

Osman Öcal, Olaf Dietrich, Sergio Lentini, Pierre Bour, Thibaut Faller, Valery Ozenne, Florian Maier, Matthias Philipp Fabritius, Daniel Puhr-Westerheide, Vanessa F. Schmidt, Elif Öcal, Ricarda Seidensticker, Moritz Wildgruber, Jens Ricke, Max Seidensticker

Table of contents

Fig. S1……………………………………………………………...…………………….2

Fig. S2……………………………………………………………..….…………………8

Supplementary video legend…………………………………………………………11

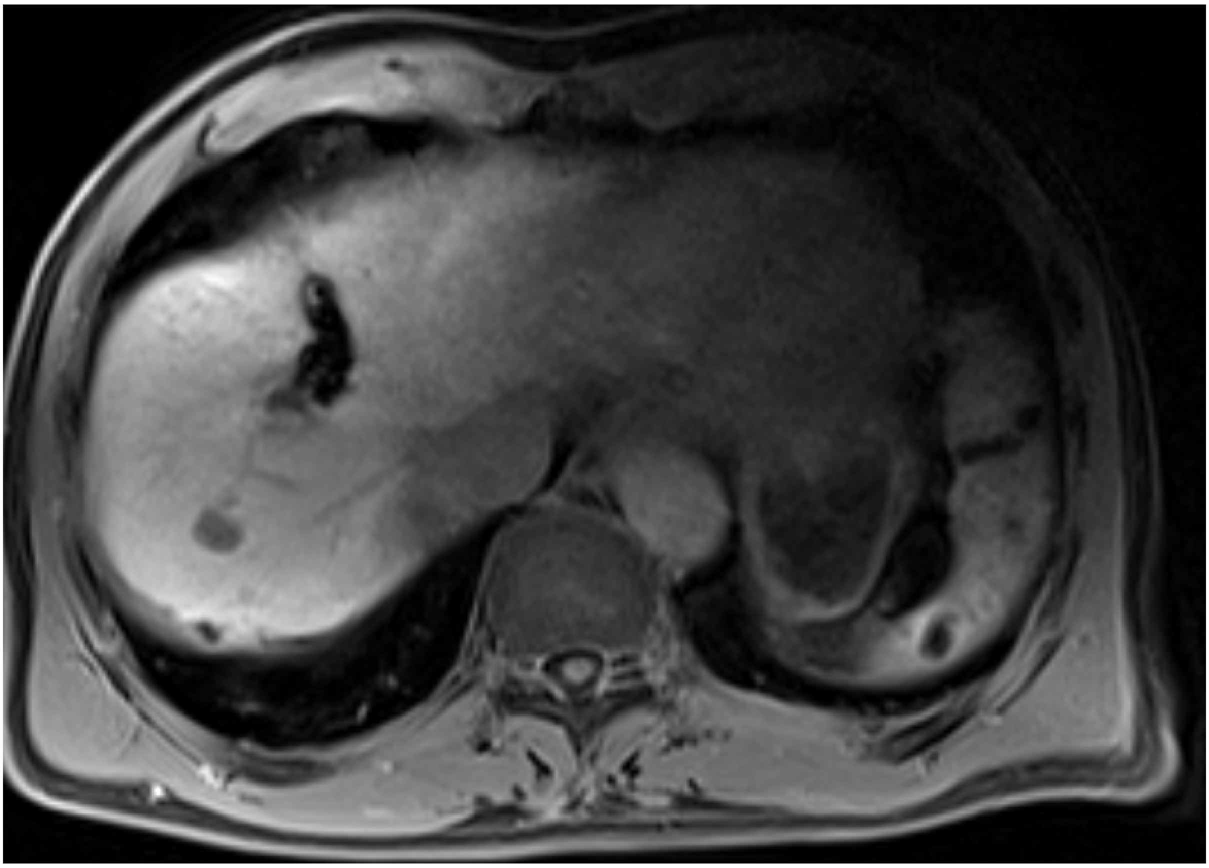

Fig. S1a. A 15x11 mm lesion is seen in Segment 7. There are artifacts related to the previous surgical materials in Segment 8.

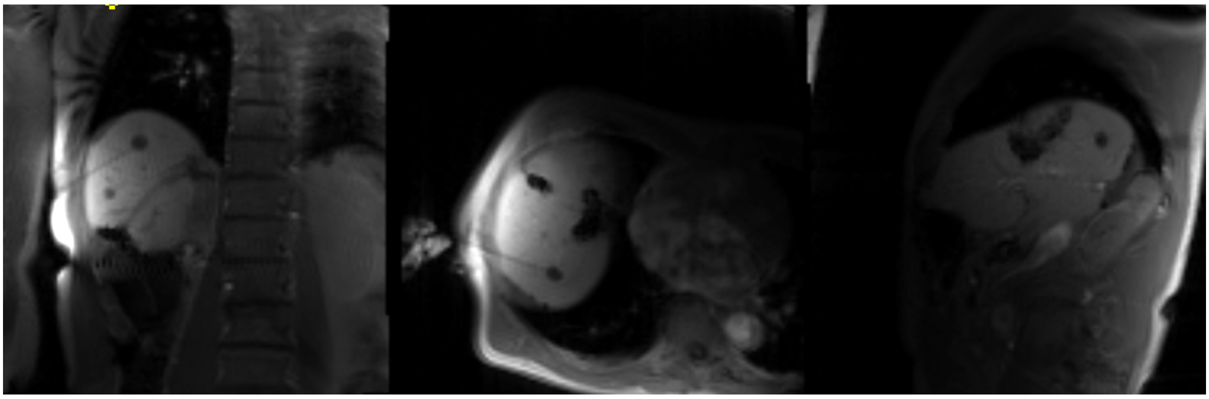

Fig. S1b. Needle placement under fluoroscopic T1w GRE sequence (BEAT interactive) in 3 perpendicular slice orientations.

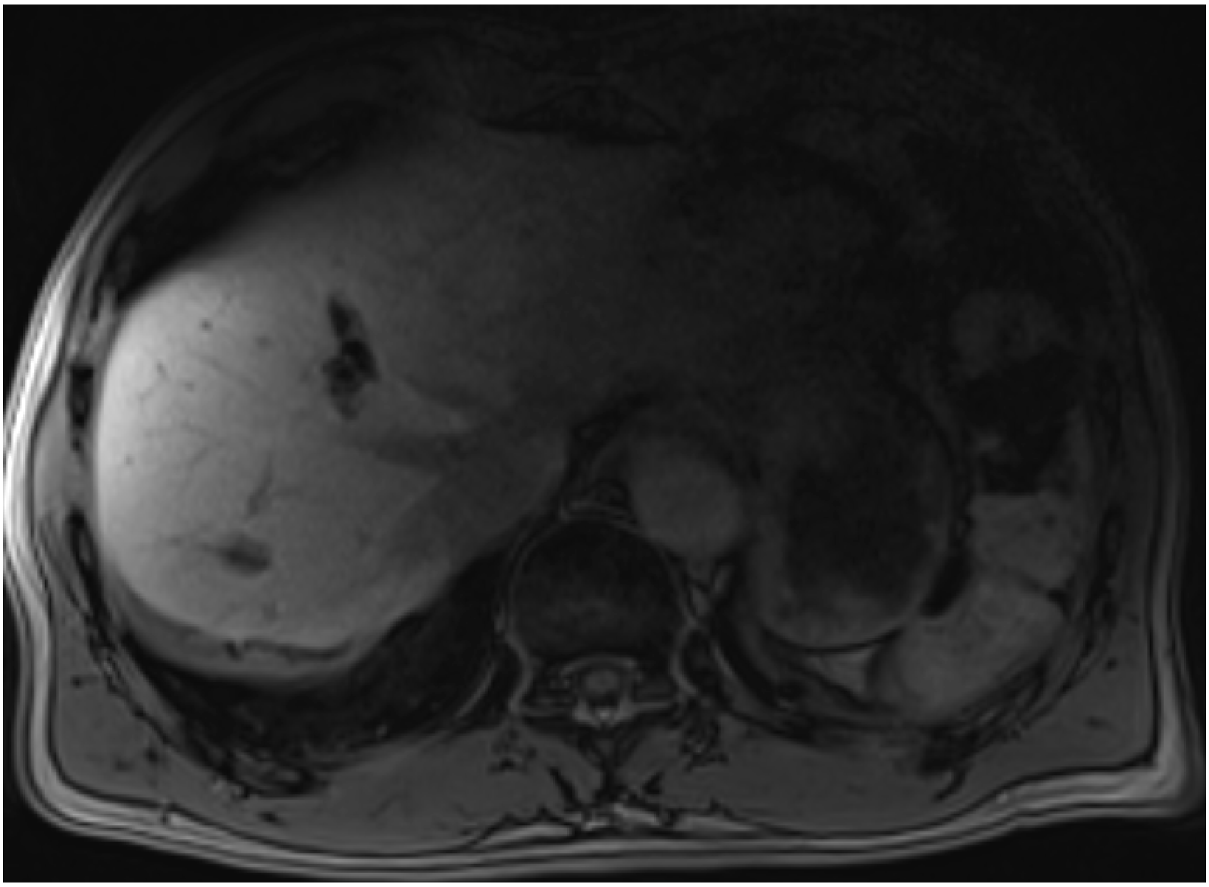

Fig. S1c. Needle position was confirmed before initation of energy deposition.

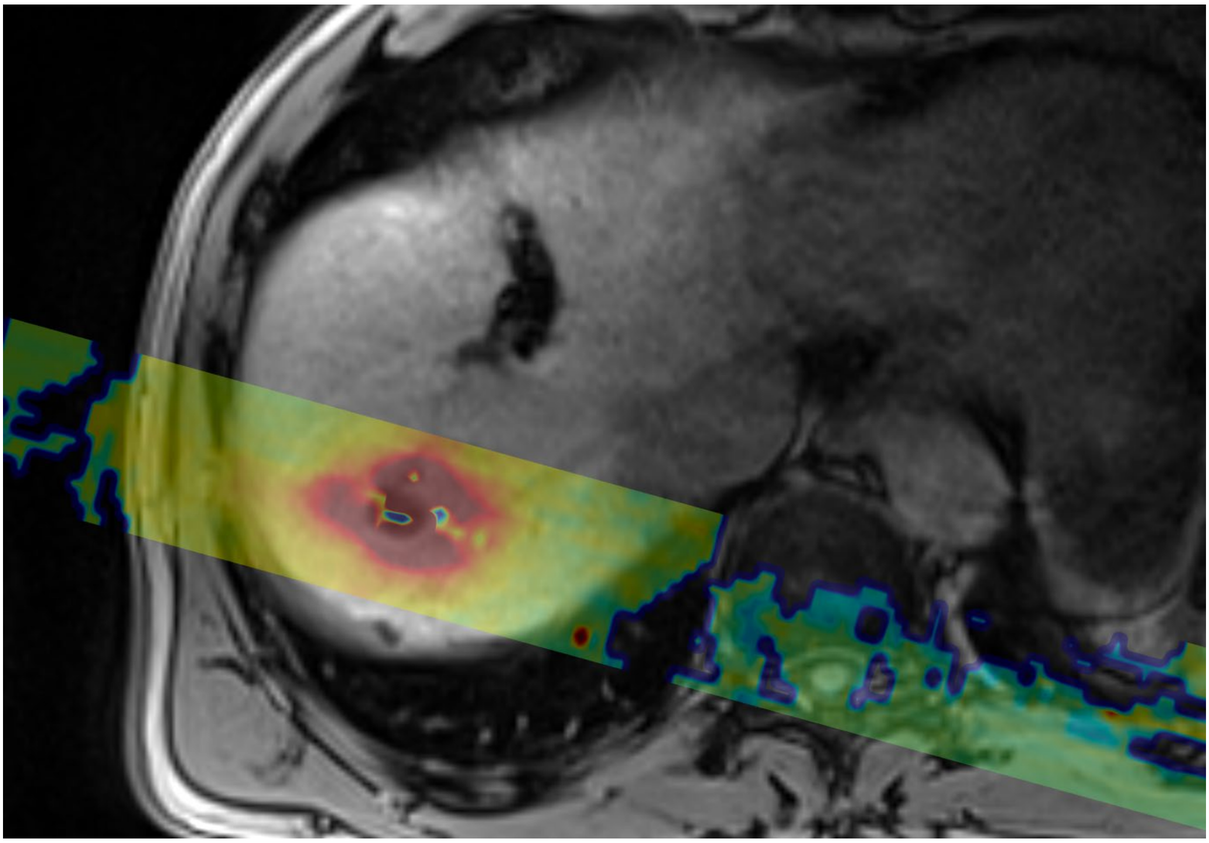

Fig. S1d. Temperature map shows satisfactory ablation volume with sufficient ablation margin in all directions.

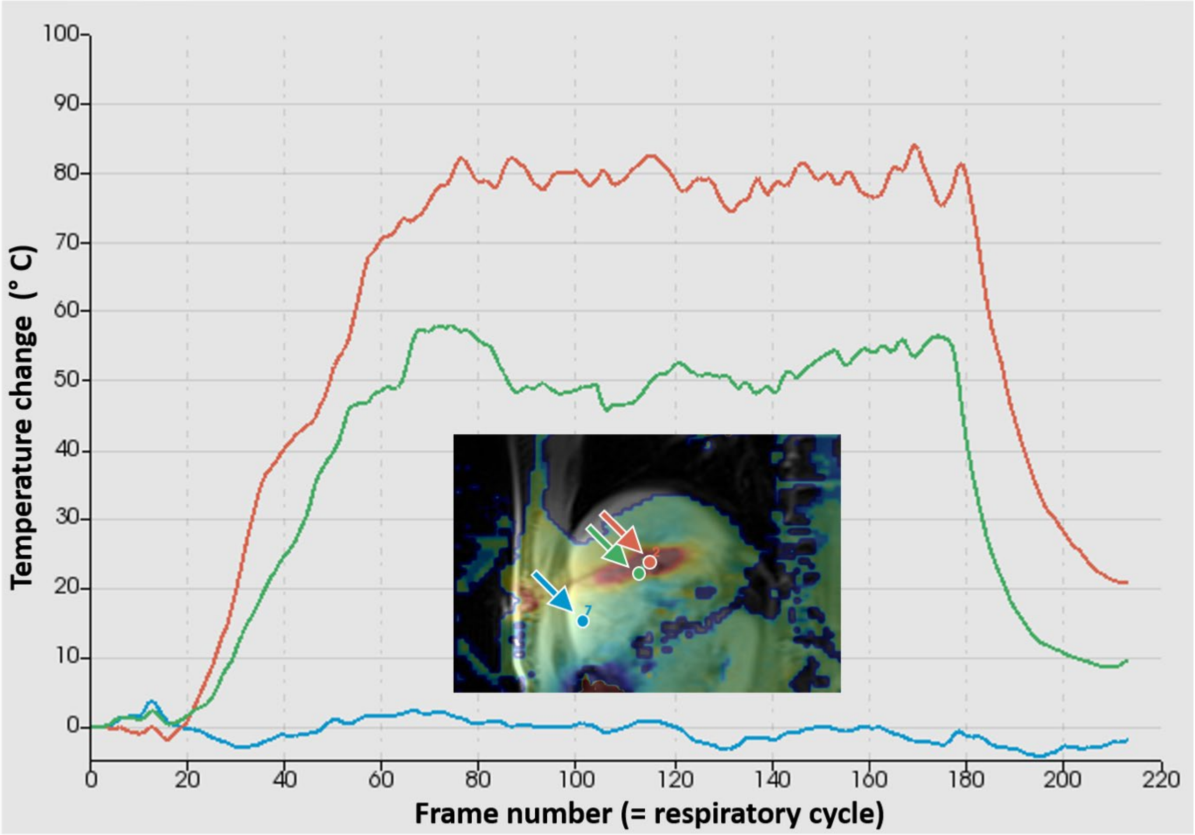

Fig. S1e. Temperature-time curve of an ablation shows temperature change at three different points within the liver. Red represents a point in the center of the lesion, green periphery, and blue a point in the liver away from the ablation area. Temperature drop within the ablation area can be seen after the completion of the energy deposition.

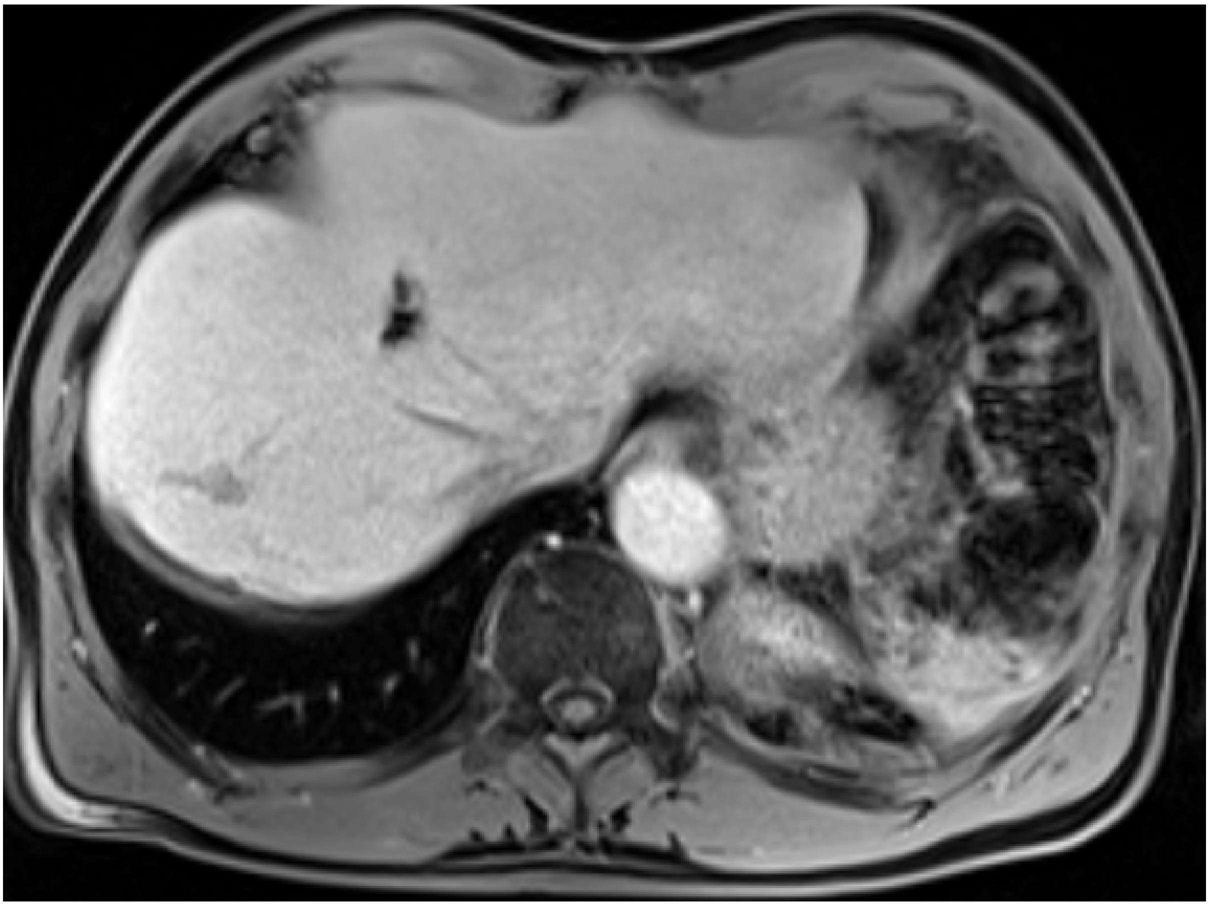

Fig. S1f. Follow-up MRI image 18 months after the treatment shows no sign of local residual disease.

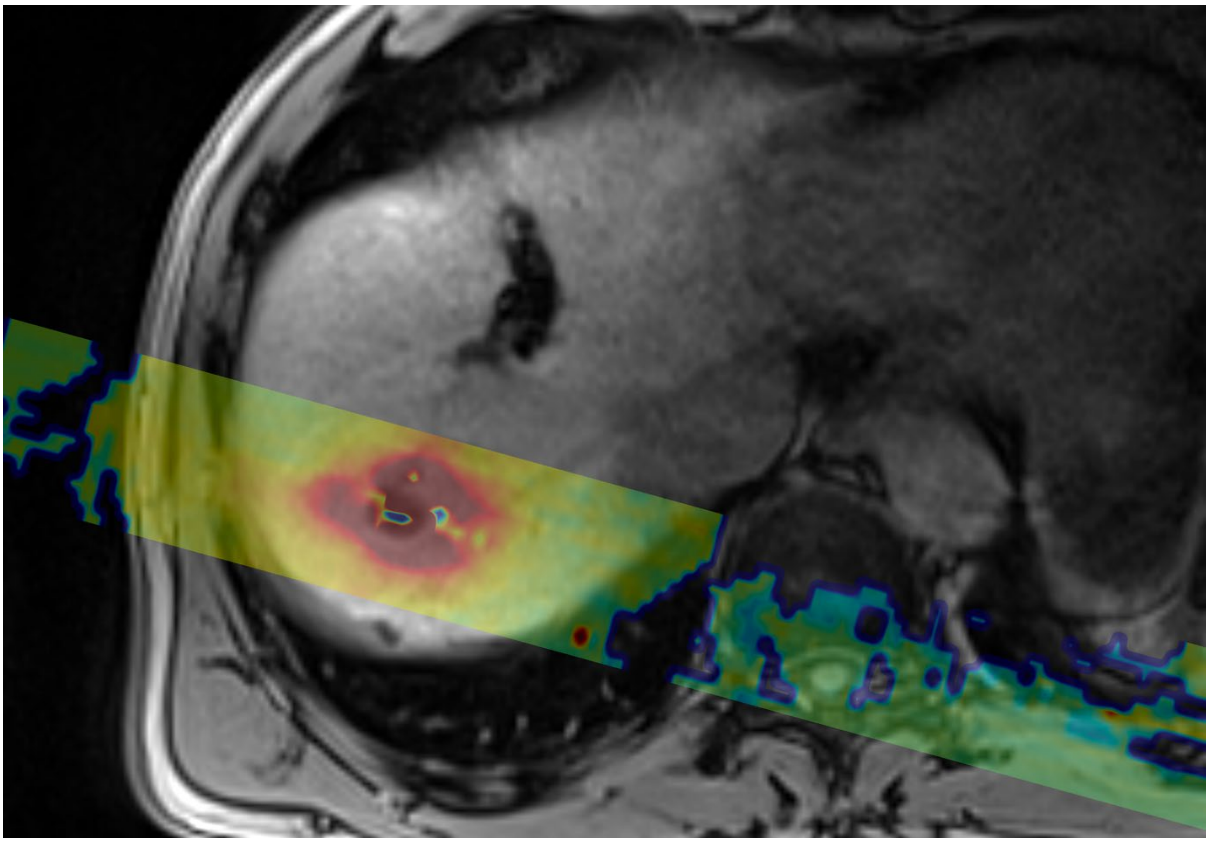

Fig. S2a. Temperature map shows no thermometry-related artifacts and no apparent movement related artifacts. The lesion can be differentiated as darker area within the red area. Both thermometry (signal-to-noise) and movement-related artifact score were graded as 1 (perfect).

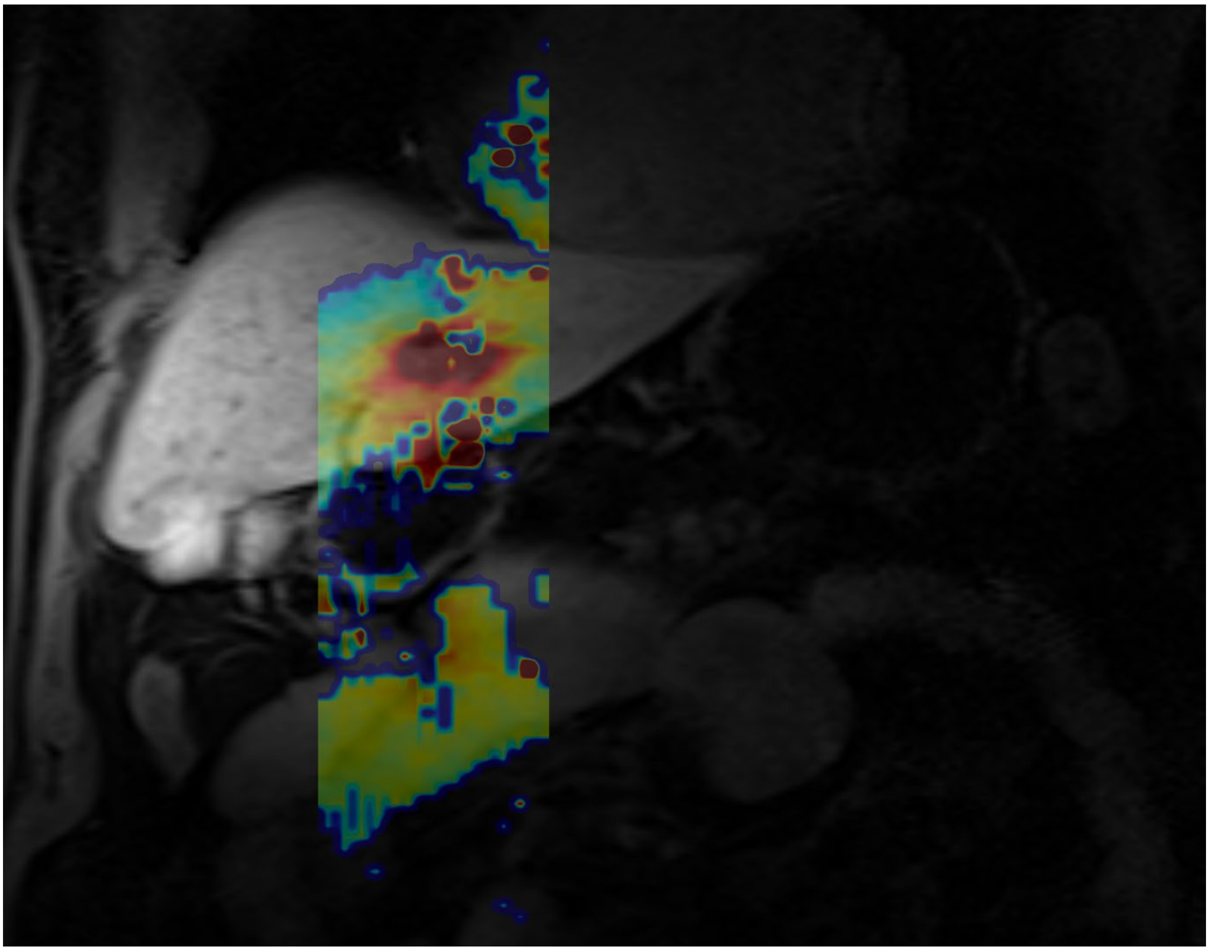

Fig. S2b. Temperature map shows good heating around the lesion, and the main thermal dose island (arrowhead) can be differentiated from movement-related artifacts reaching beyond the liver. Thermometry (signal-to-noise) score was graded as 3 and movement-related artifact as 2.

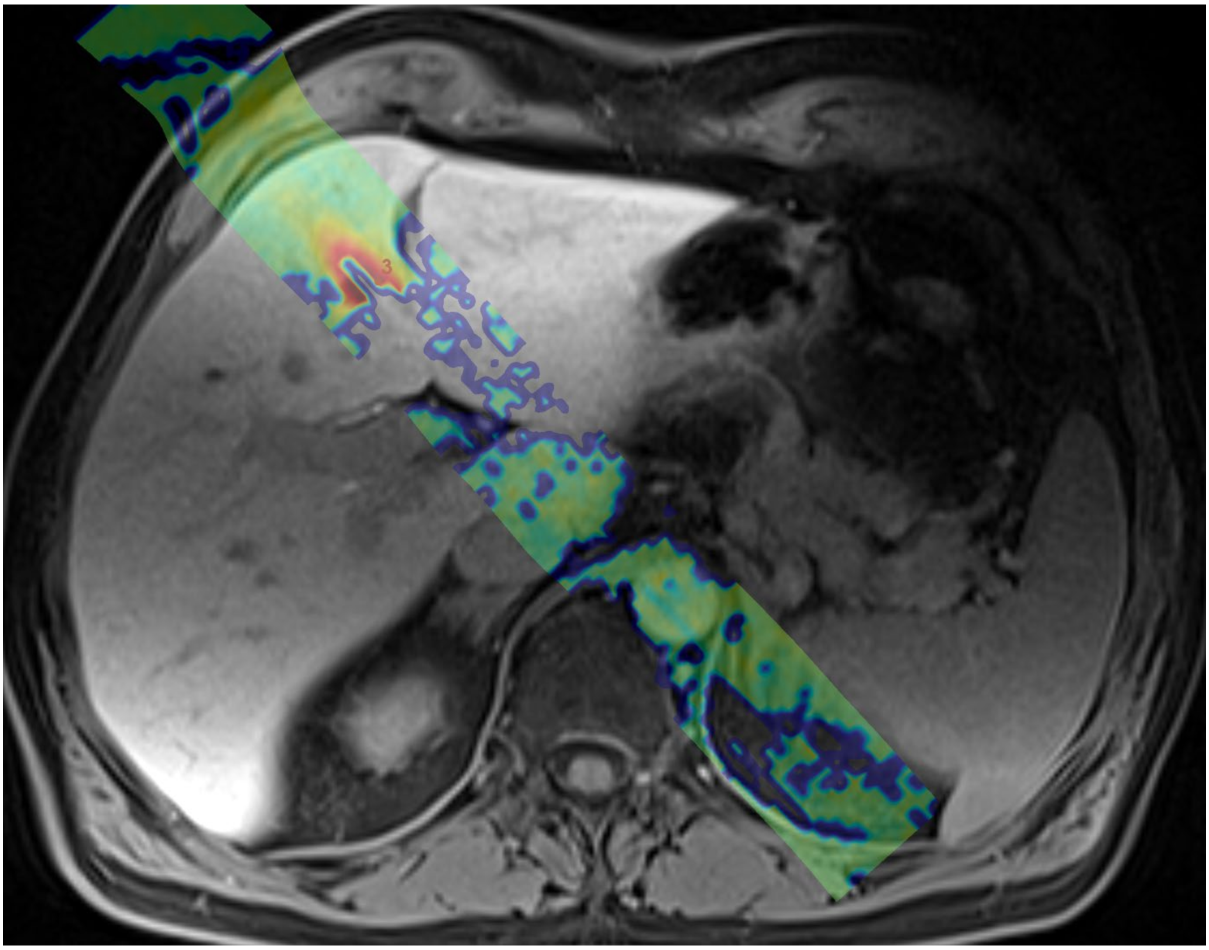

Fig. S2c. Temperature map shows heating at the superficial part of the lesion; however, no signal is received from the other parts of the lesion. The artifact related to the needle can be seen within the red area. Thermometry (signal-to-noise) score was graded as 5, and this lesion has been excluded from further analysis.

Supplementary video legend.

Real-time thermometry of an exemplary case. After the starting of energy deposition, thermometry maps visualize the temperature within the image acquisition area on a scale of green to red according to the temperature. After cessation of the energy deposition (starting from the 14th second), the temperature diffusion can be seen as shrinkage of the red areas.
